# Transcutaneous vagal nerve stimulation for treating gastrointestinal symptoms in individuals with diabetes: a randomised, double-blind, sham-controlled, multicentre trial

**DOI:** 10.1007/s00125-024-06129-0

**Published:** 2024-03-28

**Authors:** Ditte S. Kornum, Davide Bertoli, Huda Kufaishi, Anne-Marie Wegeberg, Tina Okdahl, Esben B. Mark, Katrine L. Høyer, Jens B. Frøkjær, Birgitte Brock, Klaus Krogh, Christian S. Hansen, Filip K. Knop, Christina Brock, Asbjørn M. Drewes

**Affiliations:** 1 Department of Hepatology and Gastroenterology, Aarhus University Hospital, Aarhus, Denmark; 2 Steno Diabetes Center Aarhus, Aarhus University Hospital, Aarhus, Denmark; 3 Department of Clinical Medicine, Aarhus University, Aarhus, Denmark; 4 Mech-Sense, Department of Gastroenterology and Hepatology, Aalborg University Hospital, Aalborg, Denmark; 5 Department of Clinical Medicine, Aalborg University, Aalborg, Denmark; 6 Department of Radiology, Aalborg University Hospital, Aalborg, Denmark; 7 Steno Diabetes Center Copenhagen, Herlev, Denmark; 8 Thisted Research Unit, Aalborg University Hospital Thisted, Thisted, Denmark; 9 Center for Clinical Metabolic Research, Gentofte Hospital, University of Copenhagen, Hellerup, Denmark; 10 Department of Clinical Medicine, Faculty of Health and Medical Sciences, University of Copenhagen, Copenhagen, Denmark; 11 Steno Diabetes Center North Denmark, Aalborg University Hospital, Aalborg, Denmark

**Keywords:** Autonomic neuropathy, Diabetic gastroenteropathy, Gastrointestinal dysmotility, Gastrointestinal symptoms, Gastroparesis, Heart rate variability, Vagal nerve stimulation

## Abstract

**Aims/hypothesis:**

Diabetic gastroenteropathy frequently causes debilitating gastrointestinal symptoms. Previous uncontrolled studies have shown that transcutaneous vagal nerve stimulation (tVNS) may improve gastrointestinal symptoms. To investigate the effect of cervical tVNS in individuals with diabetes suffering from autonomic neuropathy and gastrointestinal symptoms, we conducted a randomised, sham-controlled, double-blind (participants and investigators were blinded to the allocated treatment) study.

**Methods:**

This study included adults (aged 20–86) with type 1 or 2 diabetes, gastrointestinal symptoms and autonomic neuropathy recruited from three Steno Diabetes Centres in Denmark. Participants were randomly allocated 1:1 to receive active or sham stimulation. Active cervical tVNS or sham stimulation was self-administered over two successive study periods: 1 week of four daily stimulations and 8 weeks of two daily stimulations. The primary outcome measures were gastrointestinal symptom changes as measured using the gastroparesis cardinal symptom index (GCSI) and the gastrointestinal symptom rating scale (GSRS). Secondary outcomes included gastrointestinal transit times and cardiovascular autonomic function.

**Results:**

Sixty-eight participants were randomised to the active group, while 77 were randomised to the sham group. Sixty-three in the active and 68 in the sham group remained for analysis in study period 1, while 62 in each group were analysed in study period 2. In study period 1, active and sham tVNS resulted in similar symptom reductions (GCSI: −0.26 ± 0.64 vs −0.17 ± 0.62, *p*=0.44; GSRS: −0.35 ± 0.62 vs −0.32 ± 0.59, *p*=0.77; mean ± SD). In study period 2, active stimulation also caused a mean symptom decrease that was comparable to that observed after sham stimulation (GCSI: −0.47 ± 0.78 vs −0.33 ± 0.75, *p*=0.34; GSRS: −0.46 ± 0.90 vs −0.35 ± 0.79, *p*=0.50). Gastric emptying time was increased in the active group compared with sham (23 min vs −19 min, *p*=0.04). Segmental intestinal transit times and cardiovascular autonomic measurements did not differ between treatment groups (all *p*>0.05). The tVNS was well-tolerated.

**Conclusions/interpretation:**

Cervical tVNS, compared with sham stimulation, does not improve gastrointestinal symptoms among individuals with diabetes and autonomic neuropathy.

**Trial registration:**

ClinicalTrials.gov NCT04143269

**Funding:**

The study was funded by the Novo Nordisk Foundation (grant number NNF180C0052045)

**Graphical Abstract:**

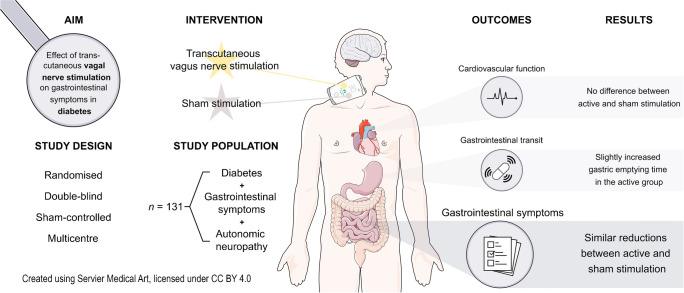


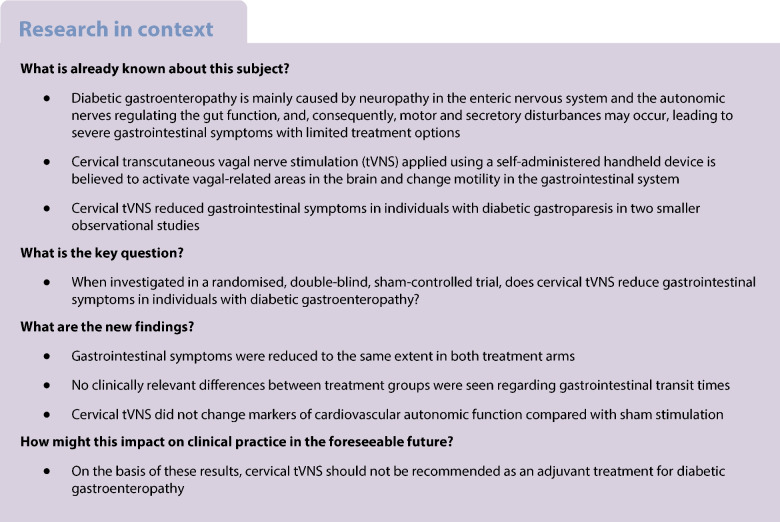


## Introduction

Diabetic gastroenteropathy affects multiple nerves, including those of the enteric nervous system and the vagal nerves, which modulate gastrointestinal function, causing widespread secretory and motor disturbances [1]. Thus, severe gastrointestinal symptoms, malnutrition, unpredictable drug absorption and poor glycaemic control may occur, significantly reducing the quality of life and increasing the disease burden [2, 3]. Progression of the underlying autonomic neuropathy may be reduced by multifactorial intervention [4], but gastrointestinal symptoms still require symptomatic management. Unfortunately, the management options have several limitations. Pharmacological therapies, e.g. prokinetic drugs, often have limited efficacy and can lead to severe side effects [5]. Surgery may be required in some individuals and may involve gastric electrical stimulation [6, 7]. The electrical stimulation improves nausea and vomiting in individuals with severe gastroparesis, probably by modulating the afferent vagal nerves without affecting gastric emptying [6, 8]. However, the procedures are invasive, carry a risk of complications, and are often inefficacious. Consequently, there is an unmet need for effective and non-invasive treatment modalities.

A potential target for future treatment is the vagal nerve, as its bidirectional signalling between the brain and the gastrointestinal system is essential for maintaining normal gastrointestinal function by modulating gastric tone etc. [9, 10]. In animal studies, transcutaneous vagal nerve stimulation (tVNS) enhances gastrointestinal motility [11]. Human studies have also demonstrated the ability of tVNS to modulate gastric motility, increase heart rate variability and activate vagal-related areas in the brain [12–14].

tVNS is a non-invasive and user-friendly treatment that primarily activates the afferent vagal nerve fibres, similar to the presumed mechanism of invasive gastric electrical stimulation [15]. Various stimulation devices are available, mainly targeting the auricular or cervical part of the vagal nerve. The cervical tVNS device gammaCore (electroCore, USA) is approved by the US Food and Drug Administration and CE-marked for treating migraine and cluster headaches [16]. Two smaller observational studies have indicated that cervical tVNS diminishes gastrointestinal symptoms in individuals with gastroparesis [17, 18]. However, randomised, double-blind, sham-controlled studies are lacking, and the symptom improvement seen in previous studies may be a placebo effect.

Diabetic gastroenteropathy affects the entire gastrointestinal tract, resulting in diverse symptoms extending beyond the stomach [19, 20]. Treatments are most often prescribed based on these clinical symptoms, as measures such as gastric emptying and segmental transit time are unsuitable for assessing treatment efficacy due to temporal variations and poor correlation with symptom severity [21].

We hypothesised that cervical tVNS improves gastrointestinal symptoms in individuals with diabetes and autonomic neuropathy. The primary aim of this study was to assess the impact of cervical tVNS on gastrointestinal symptoms. The secondary aims were to further examine the effect on gastrointestinal symptoms, gastrointestinal transit times and autonomic function.

## Methods

### Study design and participants

An investigator-initiated, double-blind, randomised, sham-controlled, multicentre trial exploring the effects of cervical tVNS on gastrointestinal symptoms in individuals with diabetes was conducted across three Steno Diabetes Centers in Denmark (North Denmark, Copenhagen and Aarhus). The participants were current patients or contacted the study personnel after reading about the study through social media or patient forums. Thus, the study included participants from both primary care and tertiary centres, covering a wide age range (20–86 years), indicating that the sample is representative of individuals with diabetes who experience gastrointestinal symptoms. Ethnicity and sex were self-reported, and sex assigned at birth was used, both reflecting the overall population composition in Denmark, with white ethnicity and female sex being slightly overrepresented. Eligible participants were >18 years old, and had a diagnosis of type 1 or type 2 diabetes confirmed at least 1 year before enrolment. The presence of gastrointestinal symptoms and verified autonomic neuropathy were required for participation. A weighted composite symptom cut-off score of 2.3 was used to ensure a significant symptom severity before inclusion. This cut-off was based on the score for the mean gastroparesis cardinal symptom index (GCSI) in four cohorts of healthy individuals plus 1 SD (0.89) and the score for the mean gastrointestinal symptom rating scale (GSRS) in three cohorts of healthy individuals plus 2 SD (1.53) [22]. The score was further weighted (GCSI ×1.33 and GSRS ×0.8) to ensure equal questionnaire contribution [23, 24]. Diabetic autonomic neuropathy was confirmed through either (1) at least one abnormal autonomic cardiovascular reflex test assessed using the VAGUS device (Medicus Engineering, Denmark) [25]; (2) a decreased sudomotor response assessed using the SUDOSCAN device (Impeto Medical, USA) with electrochemical chloride conductance cut-offs for inclusion at 50 μS for hands and 70 μS for feet [26, 27]; or (3) a score above 16 on the validated composite autonomic symptoms score (COMPASS31) [28].

Participants were required to maintain stable medication usage for 3 months before and during the study. The presence of any known concurrent gastrointestinal disease alongside diabetic gastroenteropathy led to exclusion. The complete list of exclusion criteria is available in the study protocol [22]. Baseline demographic information on height, weight, and smoking status were self-reported.

The study was approved by the North Denmark Region Committee on Health Research Ethics (N-20190020) and the Danish Medicines Agency (CIV-19-07-029105), and supervised by the independent Danish Good Clinical Practice unit, ensuring data accuracy and credibility. The study is registered at ClinicalTrials.gov (NCT04143269). All participants gave written informed consent.

### Intervention and randomisation

The study intervention was tVNS delivered using the self-administered handheld gammaCore device, stimulating the cervical part of the vagal nerve using a low-voltage electrical signal [16]. The sham device was identical to the active device but solely generated a humming sound. The study was structured in two parallel treatment groups, with participants staying in their assigned randomisation. An independent, unblinded investigator taught the participants to use the device properly and oversaw the first stimulation in each study period.

The participants were given a diary to document every stimulation in prespecified time intervals. The device was placed anterior to the sternocleidomastoid muscle, lateral to the trachea and above the carotid artery pulsation. The participants were instructed to adjust the intensity in arbitrary units from 1 to 40, using the highest tolerable but non-painful level. The tVNS was administered for 120 s bilaterally four times daily for 1 week in study period 1 and twice daily for 8 weeks in study period 2. The study periods were separated by a 2-week wash-out period [22].

Participants were randomly allocated 1:1 to receive active or sham stimulation in blocks of 8 (sealedenvelope.com). Each site received centrally allocated gammaCore and sham devices with unique anonymised numbers.

### Procedures

Gastrointestinal symptoms were assessed using two validated questionnaires. The patient assessment of upper gastrointestinal symptom severity index consists of 20 items with scores ranging from 0 (no symptoms) to 5 (very severe symptoms) [29]. The GCSI comprises nine of these questions, focusing on upper gastrointestinal symptoms divided into three categories: nausea/vomiting, bloating and postprandial fullness [23]. The GSRS was used to detect lower gastrointestinal symptoms. It comprises 15 items with scores of 1 (no discomfort) to 7 (very severe discomfort) divided into five symptom categories: reflux, abdominal pain, indigestion, diarrhoea and constipation [24]. The symptom category sub-scores represent the mean value of the included items, and the average of the mean sub-score values determines the total questionnaire score.

The SmartPill wireless motility capsule (Medtronic, USA) was used to assess segmental transit times, defined as the time it takes for the capsule to pass the stomach, small bowel, colon and the whole gut. The MotiliGI software (version 3.1) was used for data analysis, and two independent investigators analysed the data [30]. Data were compared with normative values for segmental transit times, and those exceeding the 95th percentile were categorised as prolonged [31].

Cardiac autonomic neuropathy (CAN) was evaluated using the handheld VAGUS device. The VAGUS device was used to determine whether the participants met the criteria for enrolment and it was used at baseline and after each study period [25]. Heart rate variability in response to a cardiovascular autonomic reflex test (lying-to-standing, expiration/inspiration and Valsalva manoeuvre) was registered as pathological if values fell below a predefined age-specific cut-off [32]. Based on the total score, the participant was categorised as having either absent (0), early (1) or manifest (2–3) CAN [33].

Cardiac vagal tone (CVT) representing the parasympathetic efferent activity was assessed using an eMotion Faros device (Mega Electronics, Finland) at baseline, immediately after the first stimulation (acute effect) and after each study period (long-term effects). The device is an electrocardiogram monitor that measures heart rate and phase shifts in the beat-to-beat interval during a 5 min recording with the participant resting in a seated position [34, 35]. An online application provided by ProBioMetrics (version 1.0, UK) was used to determine the CVT. CAN is indicated by a CVT value below 3.18 on a linear vagal scale where 0 represents full blockage of the parasympathetic acetylcholine neurotransmitter [34].

### Outcomes

The primary outcome measures were the changes in gastrointestinal symptoms assessed using the GCSI and the GSRS in both study periods. Secondary outcomes were gastrointestinal symptom scores adjusted for covariates, symptom category sub-scores, gastrointestinal segmental transit times, CAN score and CVT. The gastrointestinal transit times were only assessed before and after study period 2, while all other outcomes were evaluated before and after each study period.

### Statistical analysis

For each treatment group, 60 participants were required to detect a 30% difference in gastrointestinal symptoms measured by the GCSI in each study period. The trial was adequately powered according to the severity of symptoms seen in the clinic, as reported previously [22]. Participants who received at least one dose of tVNS or sham stimulation were included in the intention-to-treat analyses of the primary outcomes and adverse events. When analysing the secondary outcomes, participants who completed the study period for the specific outcome were included in per-protocol analyses.

Data were collected and handled using the electronic data management tool REDCap (https://www.project-redcap.org/). Missing data were not imputed. Baseline data are reported as means ± SD for normally distributed data, medians and IQRs for non-normally distributed data, or counts with frequencies. Compliance percentages were determined by comparing the number of registered stimulations to the total number of possible stimulations in each study period. Compliance percentages for day 1 in study period 1 were excluded because of inconsistent registration in the stimulation diary, as either first or second stimulation of the day. Primary and secondary outcomes were analysed as the change from the study period baseline in both study periods. These baseline-corrected values were compared between treatment groups using non-paired *t* tests for normally distributed data and Wilcoxon rank-sum tests for non-normally distributed data.

As a secondary outcome, multivariate linear regression analyses were used to investigate the symptom scores after each study period adjusted for the baseline symptom scores between treatment groups. The regression analyses were further adjusted for demographic characteristics, including sex, age, type and duration of diabetes and inclusion site. The model was further adjusted for compliance, baseline CAN score, CVT and gastric emptying time. The multivariate regression model was then used in a sensitivity analysis to compare participants who received active tVNS with compliance above 80% to those who received sham stimulation. Participants with a gastrointestinal symptom reduction of at least 30% in one or both questionnaires were considered responders, and were compared using a binary regression model between treatment groups. Participants exhibiting baseline symptoms beyond ‘mild symptoms’ in the GCSI or the GSRS were compared in between-group comparisons using non-paired* t* tests. Adverse events were also evaluated between groups using a binary regression model. Gastrointestinal transit times that changed from pathological to normal and vice versa were compared using a non-parametric χ^2^ test. Statistical analyses were performed using Stata version 17.0 (StataCorp, USA).

## Results

### Participants

**Fig. 1:**
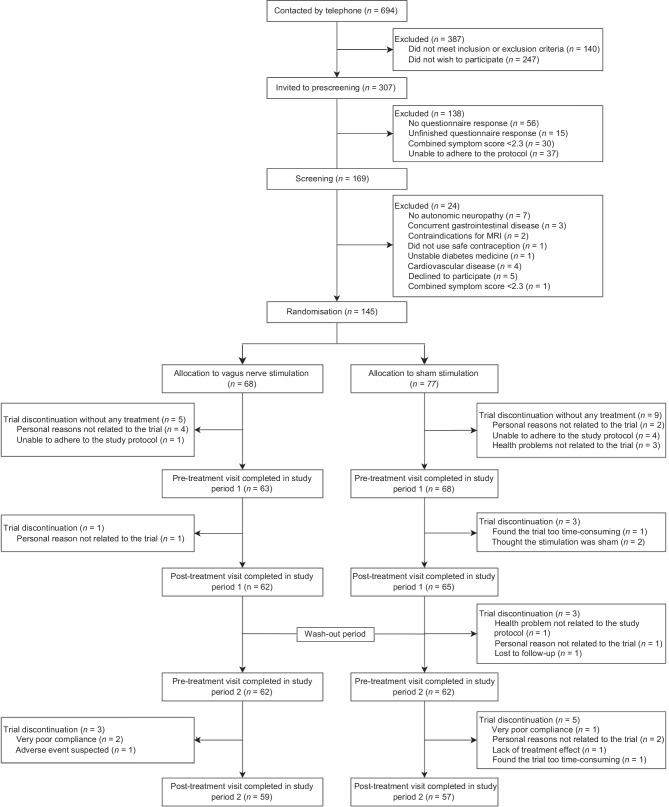
Flowchart detailing screening, randomisation and participation in both study periods

Between June 2020 and April 2022, 131 participants receiving at least one dose of tVNS were included in the intention-to-treat analyses (Fig. 1). Four of the 63 participants in the active group (6.3%) discontinued the trial before the final visit in study period 2. Overall, one person in the active group (study period 1) and three in the sham group (wash-out period and study period 2) withdrew for personal reasons. Two in the active group and one in the sham group had unacceptably poor compliance in study period 2, while two in the sham group found the trial too time-consuming (one in each study period). Furthermore, one person in the sham group had health problems (depression) (wash-out period), one was lost to follow-up (wash-out period), two thought the stimulation was a sham (study period 1), and one felt no treatment effect, leading to drop out (study period 2). Additionally, one person in the active group experienced a tVNS-related adverse event (hoarseness) in study period 2.

**Fig. 2:**
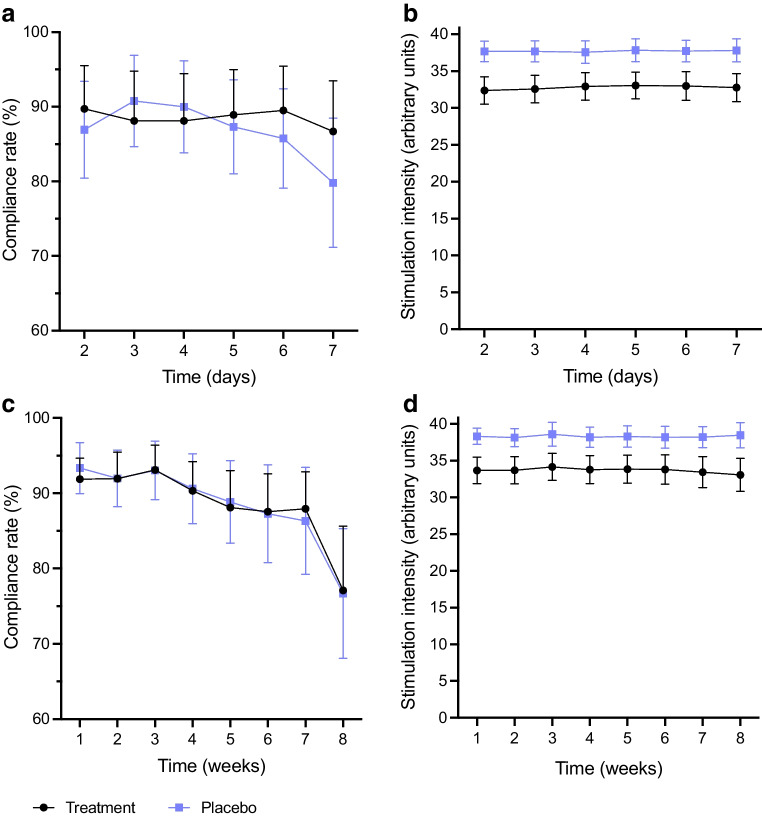
Compliance profiles (**a**, **c**) and stimulation intensity (**b**, **d**) for study period 1 (four daily stimulations for 1 week) (**a**, **b**) and study period 2 (two daily stimulations for 8 weeks) (**c**, **d**). Data are mean values with 95% CI

All participants exhibited either an abnormal CAN score or an abnormal COMPASS31 score at baseline, and none were included exclusively based on low sudomotor function. The treatment groups were comparable regarding baseline demographic, clinical and autonomic characteristics (Table 1). Compliance was 88.5% in the active group and 87.5% in the sham group for participants completing study period 1. Compliance in participants completing study period 2 was 86.3% in the active group and 85.1% in the sham group. Compliance rates for the two study periods are shown in Fig. 2.

**Table 1:** Baseline demographic, clinical and autonomic characteristics of the participants

Characteristic	Active stimulation (*n*=63)	Sham stimulation (*n*=68)
Demographic characteristics		
Age, years	54 ± 14	56 ± 14
Female	34 (54)	44 (65)
Smoking		
Current	8 (13)	10 (15)
Previous	22 (35)	29 (43)
BMI, kg/m^2^	29 ± 6	30 ± 6
White ethnicity	61 (97)	64 (94)
Clinical characteristics		
Type of diabetes		
Type 1	31 (49)	32 (47)
Type 2	32 (51)	36 (53)
Diabetes duration, years	20 (10–29)	18 (11–33)
HbA_1c_, mmol/mol	61 ± 13	62 ± 16
HbA_1c_, %	7.8 ± 1.2	7.9 ± 1.4
Charlson comorbidity index score	2 (1–2)	2 (1–3)
Systolic BP, mmHg	135 ± 15	136 ± 15
Diastolic BP, mmHg	81 ± 11	81 ± 10
Pulse, beats per min	73 ± 13^a^	73 ± 13
GCSI score	2.02 ± 1.17	1.89 ± 0.86
GSRS score	3.15 ± 1.03^a^	3.01 ± 0.92^b^
Weighted composite symptom score	5.19 ± 2.21^a^	4.94 ± 1.71^b^
Autonomic characteristics		
COMPASS31 score	35.36 ± 15.64	39.89 ± 15.75
COMPASS31 score >16	54 (86)	62 (91)
CAN score^c^		
No CAN	22 (38)	31 (48)
Early CAN	14 (24)	19 (29)
Manifest CAN	22 (38)	15 (23)
SUDOSCAN results		
ECC for the hands, µS	60 (44–74)	58 (42–69)
ECC for the hands <50µS	19 (30)	24 (35)
ECC for the feet, µS	76 (52–84)	72 (57–82)
ECC for the feet <70µS	26 (41)	31 (46)
CVT	2.97 (1.89–4.49)	2.72 (1.72–5.64)
CVT <3.18	31 (49)	35 (52)
Transit times		
Gastric emptying time, h:min	3:20 (2:45–5:01)	3:21 (2:31–4:28)
Long gastric emptying time	15 (24)	12 (18)
Small-bowel transit time, h:min	4:52 (3:44–5:51)	4:27 (3:21–5:07)
Long small-bowel transit time	10 (16)	9 (13)
Colonic transit time, h:min	37:05 (17:28–60:48)	33:48 (17:49–64:48)
Long colonic transit time	17 (27)	15 (22)
Whole-gut transit time, h:min	46:50 (18:00–76:50)	46:50 (23:00–74:00)
Long whole-gut transit time	16 (25)	14 (21)

Data are means ± SD, medians (IQR) or *n* (%)

A long gastric emptying time is defined as >4:58 (women) or >4:53 (men). A long small-bowel transit time is defined as >8:42 (women) or >5:45 (men). A long colonic transit time is defined as >49:37 (women) or >50:32 (men). A long whole-gut transit time is defined as >72:40 (women) or >65:28 (men)

^a^=62; ^b^*n*=66; ^c^*n*=58 (active group) and *n*=65 (sham group)

ECC, electrochemical chloride conductance

### Primary outcomes: gastrointestinal symptoms

**Fig. 3:**
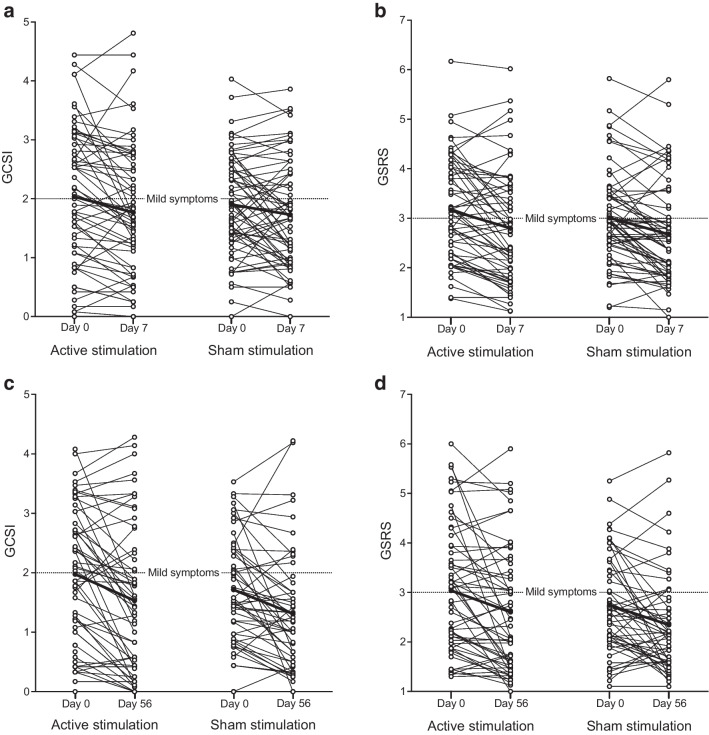
Mean values for the GCSI (**a**, **c**) and the GSRS (**b**, **d**) visualised before and after study period 1 (**a**, **b**) and study period 2 (**c**, **d**). The thicker lines represent the mean values for each treatment arm

In study period 1, there was no difference in symptom score reductions between the treatment groups (Fig. 3). The GCSI reduction was −0.26 ± 0.64 in the participants receiving active tVNS (mean ± SD) and −0.17 ± 0.62 in the sham group (*p*=0.44) (Table 2). Similarly, the GSRS decreased by −0.35 ± 0.62 after active stimulation and by −0.32 ± 0.59 in the sham group (*p*=0.77). There were no differences in symptom scores between the groups in study period 2. The GCSI decreased by −0.47 ± 0.78 after active stimulation and by −0.33 ± 0.75 in the sham group (*p*=0.34). The mean GSRS symptom reduction was −0.46 ± 0.90 in the active group compared with −0.35 ± 0.79 in the sham group (*p*=0.50).

**Table 2:** Changes in primary and secondary outcomes for each study period

Characteristic	Study period 1					Study period 2				
	Active stimulation	*N*	Sham stimulation	*N*	*p* value	Active stimulation	*N*	Sham stimulation	*N*	*p* value
GCSI	−0.26 ± 0.64	63	−0.17 ± 0.62	65	0.44	−0.47 ± 0.78	56	−0.33 ± 0.75	52	0.34
Nausea/vomiting score	0.00 (−0.33 to 0.33)	63	0.00 (−0.33 to 0.34)	65	0.84	−0.33 (−0.67 to 0.00)	56	−0.33 (−0.67 to 0.00)	52	0.75
Bloating score	−0.5 (−1.00 to 0.00)	63	0.00 (−1.00 to 0.00)	65	0.62	−0.5 (−1.50 to 0.00)	56	0.00 (−1.00 to 0.00)	52	0.09
Fullness score	−0.25 (−0.75 to 0.00)	63	0.00 (−0.75 to 0.25)	65	0.21	−0.25 (−1.00 to 0.00)	56	−0.25 (−0.75 to 0.38)	52	0.29
GSRS	−0.35 (0.62)	62	−0.32 (0.59)	64	0.77	−0.46 (0.90)	58	−0.35 (0.79)	53	0.50
Reflux score	0.00 (−0.50 to 0.00)	62	0.00 (−0.75 to 0.25)	64	0.58	0.00 (−1.00 to 0.00)	58	0.00 (−0.50 to 0.00)	53	0.89
Abdominal pain score	0.00 (−0.67 to 0.00)	62	−0.33 (−1.00 to 0.33)	64	0.53	−0.33 (−1.00 to 0.00)	58	−0.33 (−0.67 to 0.33)	53	0.54
Indigestion score	−0.38 (−1.00 to 0.00)	62	−0.38 (−1.00 to 0.00)	64	0.78	−0.25 (−1.00 to 0.00)	58	−0.50 (−1.00 to 0.00)	53	0.93
Diarrhoea score	−0.34 (−1.33 to 0.00)	62	−0.33 (−0.84 to 0.33)	64	0.14	0.00 (−0.67 to 0.34)	58	0.00 (−1.00 to 0.67)	53	0.83
Constipation score	−0.33 (−1.00 to 0.00)	62	−0.34 (−1.00 to 0.00)	64	0.96	−0.33 (−1.00 to 0.00)	58	0.00 (−1.00 to 0.34)	53	0.52
Combined weighted symptom score	−0.64 ± 1.16	62	−0.51 ± 1.11	64	0.51	−0.99 ± 1.61	56	−0.72 ± 1.49	52	0.37
Changes in CVT										
Acute effect	0.25 (−0.09 to 0.72)	54	0.35 (−0.15 to 1.10)	56	0.42	0.03 (−0.53 to 0.49)	52	0.04 (−0.46 to 0.59)	47	0.93
Long-term effect	0.06 (−0.66 to 0.57)	48	0.06 (−0.93 to 0.58)	51	0.83	−0.17 (−1.15 to 0.56)	47	0.24 (−0.72 to 0.86)	41	0.14
Changes in the CAN score										
Lying-to-standing test	0.00 (−0.02 to 0.04)	54	0.00 (−0.04 to 0.02)	56	0.07	0.00 (−0.04 to 0.03)	54	0.00 (−0.05 to 0.02)	51	0.42
Expiration/inspiration test	−0.01 (−0.02 to 0.03)	57	−0.02 (−0.06 to 0.02)	56	0.02	0.00 (−0.03 to 0.02)	53	−0.01 (−0.03 to 0.03)	51	0.99
Valsalva manoeuvre test	0.01 (−0.10 to 0.16)	35	0.01 (−0.07 to 0.10)	45	0.97	−0.03 (−0.10 to 0.06)	37	0.01 (−0.05 to 0.08)	39	0.32
Changes in transit times										
Gastric emptying time, min						23 (−36 to 116)	46	−19 (−90 to 93)	49	0.04
Small-bowel transit time, min						−7.0 (−65 to 47)	46	4.5 (−38 to 45)	50	0.57
Colonic transit time, min						69 (−1416 to 967)	46	60 (−451 to 744)	50	0.65
Whole-gut transit time, min						82 (−1419 to 1314)	46	79 (−646 to 922)	49	0.87

Data are means ± SD or medians (IQR)

Data are baseline-corrected values for study periods 1 and 2. Thus a negative value represents a reduction in the outcome variable across the stimulation period and a positive value represents an increase

Although it was not a primary outcome, treatment responses were analysed. In study period 1, 21 of 63 participants (33.3%) in the active group were responders, compared with 23 of 68 participants (33.8%) in the sham group (RR 0.99; 95% CI 0.61, 1.60; *p*=0.95). In study period 2, 26 of 62 participants (41.9%) in the active group were responders, compared with 24 of 62 participants (38.7%) in the sham group (RR 1.08; 95% CI 0.71, 1.66; *p*=0.71).

### Secondary outcomes: gastrointestinal symptoms

The multivariate linear regression analyses confirmed similar symptom changes between treatment groups when adjusting for demographic covariates. Additionally, adjusting for compliance, baseline values for the CAN score, CVT and gastric emptying time did not affect the difference in symptom scores between groups (Table 3). Sensitivity analyses comparing participants in the active group who showed compliance >80% with those in the sham group showed similar symptom score differences when adjusting for the same covariates (Table 4).

**Table 3:** Multiple linear regression analysis of the symptom scores after each study period comparing the active group with the sham group

Symptom score	Model 1		Model 2		Model 3	
	Mean difference (95% CI)	*p* value	Mean difference (95% CI)	*p* value	Mean difference (95% CI)	*p* value
Study period 1						
GCSI	−0.06 (−0.27, 0.15)	0.54	−0.07 (−0.28, 0.14)	0.51	0.10 (−0.18, 0.39)	0.46
GSRS	−0.02 (−0.23, 0.19)	0.87	−0.03 (−0.24, 0.19)	0.81	0.11 (−0.16, 0.38)	0.42
Combined weighted symptom score	−0.11 (−0.51, 0.29)	0.59	−0.13 (−0.53, 0.28)	0.54	0.22 (−0.30, 0.74)	0.41
Study period 2						
GCSI	−0.09 (−0.38, 0.20)	0.55	−0.11 (−0.41, 0.18)	0.50	−0.05 (−0.41, 0.31)	0.59
GSRS	−0.02 (−0.32, 0.29)	0.92	−0.01 (−0.31, 0.29)	0.95	−0.00 (−0.37, 0.36)	0.99
Combined weighted symptom score	−0.16 (−0.75, 0.43)	0.59	−0.18 (−0.76, 0.40)	0.55	−0.11 (−0.83, 0.61)	0.77

Data are mean symptom score differences between treatment groups (95% CI)

Model 1 comprised baseline symptom score adjustment. Model 2 comprised baseline symptom score and demographic adjustments. Model 3 comprised baseline symptom score, demographic, autonomic and compliance adjustments

**Table 4:** Multiple linear regression analysis of the symptom scores after each study period in participants receiving active vagal nerve stimulation with compliance above 80% compared with those receiving sham stimulation

Symptom score	Model 1		Model 2		Model 3	
	Mean difference (95% CI)	*p* value	Mean difference (95% CI)	*p* value	Mean difference (95% CI)	*p* value
Study period 1						
GCSI	−0.11 (−0.33, 0.12)	0.34	−0.12 (−0.35, 0.12)	0.30	0.17 (−0.14, 0.48)	0.28
GSRS	−0.08 (−0.30, 0.13)	0.45	−0.10 (−0.32, 0.13)	0.40	0.05 (−0.23, 0.33)	0.72
Combined weighted symptom score	−0.20 (−0.62, 0.22)	0.35	−0.23 (−0.67, 0.20)	0.29	0.23 (−0.35, 0.80)	0.43
Study period 2						
GCSI	−0.14 (−0.44, 0.17)	0.38	−0.18 (−0.49, 0.13)	0.25	−0.19 (−0.60, 0.21)	0.35
GSRS	−0.07 (−0.40, 0.25)	0.65	−0.10 (−0.41, 0.22)	0.54	−0.14 (−0.54, 0.27)	0.51
Combined weighted symptom score	−0.26 (−0.89, 0.36)	0.41	−0.33 (−0.94, 0.29)	0.29	−0.39 (−1.20, 0.43)	0.34

Data are mean symptom score differences between treatment groups (95% CI)

Ten participants were excluded in study period 1, and 13 were excluded in study period 2 because of compliance below 80%

Model 1 comprised baseline symptom score adjustment. Model 2 comprised baseline symptom score and demographic adjustments. Model 3 comprised baseline symptom score, demographic, autonomic and compliance adjustments

**Fig. 4:**
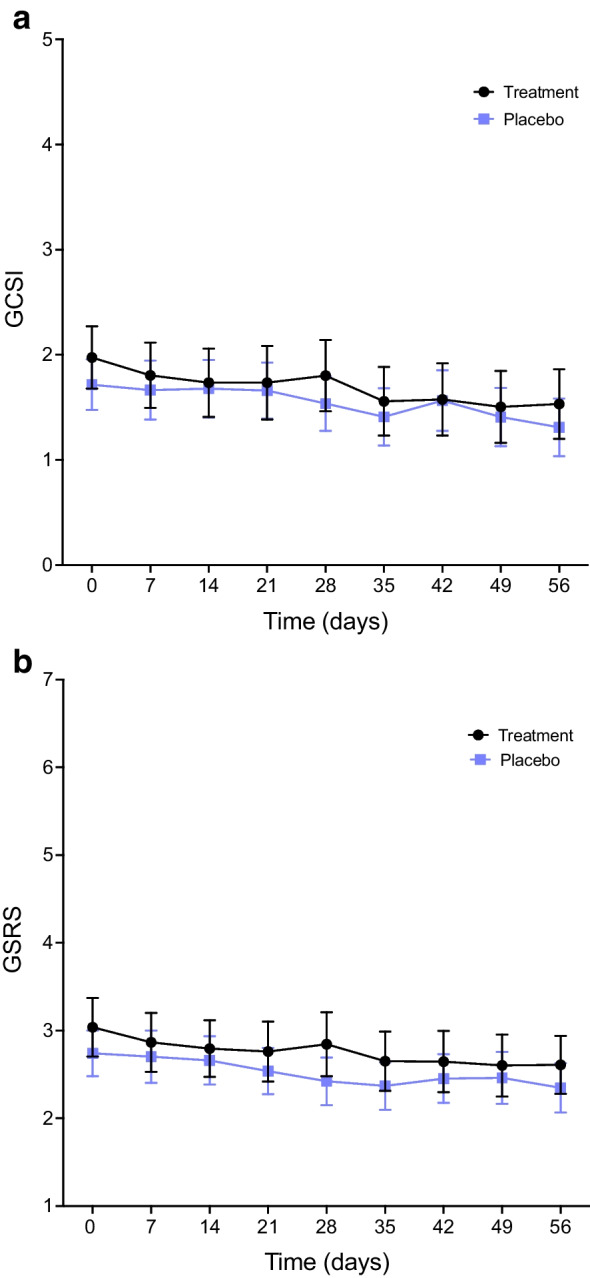
The mean GCSI (**a**) and GSRS (**b**) for each week in study period 2. Data are mean values with 95% CI

Figure 4 presents the GCSI and GSRS scores assessed weekly during study period 2, with no differences being observed between the treatment groups. Changes in symptom category sub-scores from each questionnaire were comparable between groups (Table 2). No differences were seen between the groups during either study period when analysing the symptom change in only participants with a baseline GCSI >2 or GSRS >3 (all *p*>0.50).

### Secondary outcomes: gastrointestinal transit times

**Fig. 5:**
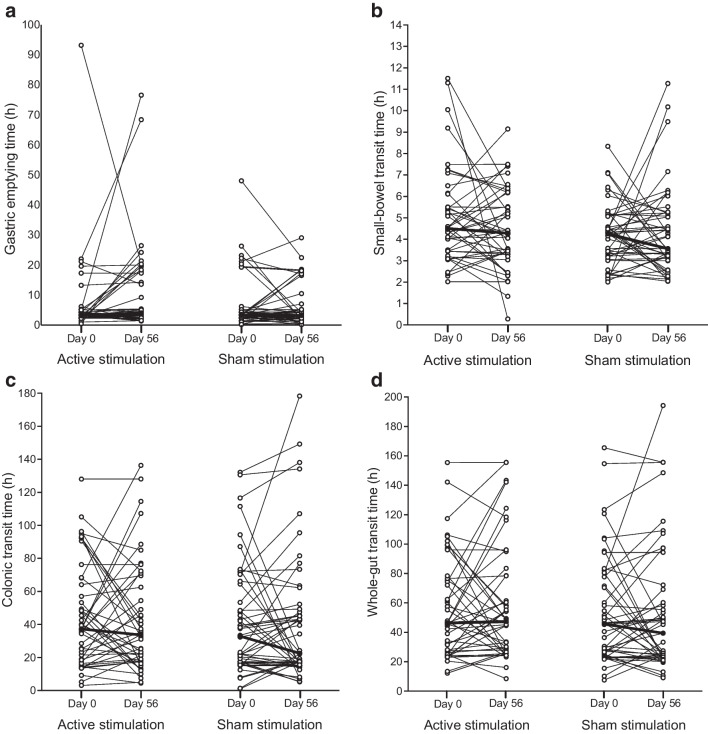
Median gastric (**a**), small-bowel (**b**), colonic (**c**) and whole-gut (**d**) transit times before and after the 8 weeks of two daily stimulations in study period 2. The thicker lines represent the median values for each treatment arm

Transit times measured before and after study period 2 are presented in Fig. 5. In the active group, one participant with a gastric emptying time that was over 10 SD above the mean value was excluded from the analysis. A median gastric emptying time increase of 23 min (IQR −36 to 116 min) was observed in the active group, while the sham group experienced a median decrease of −19 min (IQR −90 to 93 min) (*p*=0.04). No differences were seen when comparing the small-bowel, colonic or whole-gut transit times (Table 2). The proportions of transit time measurements that changed from pathological to normal were comparable between treatment groups (all *p* values >0.16).

### Secondary outcomes: autonomic function

Table 2 provides information on cardiovascular autonomic measurements. No differences were seen in the sub-scores that comprise the CAN score, except for the inspiration/expiration ratio in study period 1, where the sham group showed a higher median decrease (−0.02; IQR −0.06 to 0.02) than the active group (−0.01; IQR −0.02 to 0.03) (*p*=0.02). Neither acute nor long-term changes in CVT were observed.

### Safety

Sixteen participants (25.4%) receiving active tVNS and 13 participants (19.1%) receiving sham stimulation had at least one adverse event (RR 1.36; 95% CI 0.70, 2.61; *p*=0.36). These numbers include the one participant experiencing hoarseness after active tVNS, which led to trial discontinuation. Otherwise, the active group mainly experienced tension headaches and muscular discomfort at the stimulation site, while five participants in each group reported increased gastrointestinal complaints.

## Discussion

To our knowledge, this is the first randomised, double-blind, sham-controlled trial assessing the clinical effect of tVNS on gastrointestinal symptoms in individuals with diabetes and autonomic neuropathy. The active and sham stimulation similarly reduced gastrointestinal symptoms during a short-term high-intensity stimulation period and a long-term medium-intensity stimulation period. A minor increase was seen for gastric emptying in the active group, but otherwise, segmental gastrointestinal transit times, CAN score and CVT were similar between the treatment arms.

### Potential mode of action of vagal neuromodulation

The gammaCore device is optimised to induce signals in the afferent myelinated Aβ vagal nerve fibres [16]. Assessing somatosensory evoked brain potentials and performing functional magnetic resonance scans of the brain and brainstem during cervical tVNS has demonstrated the activation of vagal fibres in healthy individuals [14, 36, 37]. Studies in animals and healthy individuals have shown that tVNS modulates fundamental patterns of gastrointestinal motility and increases heart rate variability measurements [11–13, 38, 39]. Thus, tVNS is believed to stimulate afferent vagal nerve fibres, transmitting the signals to the brainstem and the brain, where an efferent vagal nerve signal is conveyed to the gastrointestinal system [40].

### Effect of tVNS on gastrointestinal symptoms

There is a lack of standardisation for tVNS regarding the intensity, frequency, pulse duration, length, anatomical site and type of device. As a result, it is challenging to compare results across studies [41]. Two smaller observational, non-randomised, uncontrolled studies investigated the effect of cervical tVNS in individuals with medically refractory and severely symptomatic gastroparesis of various aetiologies, including diabetes [17, 18]. Both studies reported a decreased symptomatic burden in 35–40% of the participants. The present trial shows a comparable proportion of responders in both treatment arms, stressing the importance of having a placebo group. Our participants are different from those in the above-mentioned studies. First, we only included participants with diabetes. Second, a verified gastroparesis diagnosis was not a criterion for inclusion as this measure correlates poorly with symptoms [6] and shows large intra-study variability (see below). Third, as diabetic gastroenteropathy is a pan-enteric condition, we included patients with a broader spectrum of symptoms than just the cardinal symptoms of gastroparesis. In the present study, most participants also complained of upper gastrointestinal symptoms as evaluated by the overall GCSI and by the symptom category sub-scores for nausea/vomiting, bloating and fullness. The bloating and fullness sub-scores were the primary drivers for the symptom decrease in the active group but no difference in these sub-scores was seen when comparing the treatment groups.

The cut-off value defining a symptomatic response to a given treatment lacks consensus. We defined treatment success as a 30% decrease in one or both symptom scores. This threshold was chosen to reflect the fact that a higher baseline symptom score may lead to a more prominent symptom reduction than a lower baseline score. The proportion of responders increased twofold when applying the 0.75-point GCSI cut-off used in the above-mentioned observational studies. However, the proportion of responders in the active and sham groups remained comparable. A randomised, double-blind pilot study has investigated the effect of cervical tVNS on lower gastrointestinal symptoms in 19 individuals with Parkinson’s disease and gastrointestinal complaints. It showed a GSRS symptom reduction in the active arm, but no differences were observed when comparing the post-treatment symptom scores between the active and sham groups [42]. In line with this study, we also observed a degree of GSRS symptom reduction across treatment groups.

Anatomical variations between men and women, such as differences in subcutaneous adipose tissue, muscular density and subjective compliance, may influence these outcomes. Therefore, sex was included as a covariate in the multiple linear regression analyses, but it did not alter the overall conclusions, and the results are most likely applicable to both sexes in the population.

### Effect of tVNS on gastrointestinal transit times

Most studies have not demonstrated changes in gastric emptying following the implantation of gastric neurostimulators [7]. In contrast, we found a minor increase in gastric emptying time in the active group, but no changes were seen for small-bowel or colonic transit times. A longer gastric emptying time has the potential to increase symptoms such as nausea and vomiting. Thus, these findings contradicted the anticipated outcome of the tVNS treatment.

We observed quite large intra-individual and inter-individual transit time variations, especially for gastric emptying time, which are unsurprising. Previous studies have demonstrated that individuals with diabetes and upper gastrointestinal symptoms have more significant transit time fluctuations than healthy individuals [21]. One study investigated diabetic and non-diabetic individuals with gastrointestinal symptoms. It showed that, across two subsequent measurements 14–16 days apart, 30% of the participants had a different gastric emptying time (normal, more rapid or delayed) [43]. This is in line with the present study, in which 24% of participants in the active group and 31% in the sham group had different gastric emptying times (pathological or normal) when comparing assessments before and after study period 2.

### Effect of tVNS on cardiac autonomic function

In the present study, tVNS changed neither the CAN score nor the CVT. In healthy individuals, tVNS was shown to increase CVT [13]. In individuals with chronic pancreatitis, a study showed increased CVT when combining tVNS with deep, slow breathing, but another study showed no effect [44, 45]. In individuals with gastroparesis of various aetiologies, cervical tVNS did not improve heart rate variability [18]. In the present study, the median CVT was in the lowest part of the normal range and below the cut-off for recognising established CAN [34, 35]. Furthermore, more than 50% of the participants had an abnormal CAN score at baseline, representing early or manifest CAN. Thus, tVNS may not effectively induce a sufficient efferent nervous signal to change the heart rate variability in individuals with autonomic neuropathy.

### Strengths and limitations of the study

The present study has notable strengths, including examining short-term high-intensity and long-term medium-intensity tVNS using a randomised, sham-controlled and double-blind design. The involvement of multiple centres allowed inclusion of more participants, further strengthening the study. Additionally, compliance levels were high and consistent across the active and sham groups.

There are also several limitations. First, the tVNS was self-administered without real-time assessment of vagal nerve fibre activation. Therefore, it is impossible to directly determine the ‘exposure’ to the active stimulation. Second, the intended vagal modulation may be hampered by autonomic neuropathy in any of the vagal neurocircuits within the brainstem, midbrain and higher cortical centres controlling gastrointestinal motility [46]. Third, no established standards exist for stimulation duration and daily treatment frequency for treating gastrointestinal symptoms. However, the dosage of two consecutive 120 s stimulations twice daily has proven effective as a prophylactic approach for treating primary headaches [16, 47]. Previous studies targeting gastrointestinal symptoms have often used two to four daily stimulations [17, 18, 42]. Long-term treatment compliance may be limited by the time-consuming nature of the handheld application, especially with treatment frequencies exceeding two daily stimulations. Fourth, the participants had either type 1 or type 2 diabetes, reducing the homogeneity of the cohort. However, studies have shown a comparable prevalence of diabetic autonomic neuropathy between the diabetes categories [6]. Adjusting for diabetes type in the multiple linear regression analyses did not affect the symptom score difference between treatment arms. Fifth, autonomic neuropathy was determined using either the COMPASS31 questionnaire to assess autonomic symptoms, the VAGUS device to evaluate cardiovascular autonomic reflex test, or the SUDOSCAN device to estimate sudomotor function by measuring electrochemical skin conductance. These methods are all broadly accessible and easy to use. While more accurate methods exist, they are more complex and time consuming, making them unsuitable for this study [33, 48]. In addition, the SUDOSCAN measurement is only a surrogate measure of autonomic function, and the sweat response may not be exclusively induced by sympathetic autonomic fibres [49]. Sixth, measures of transit times were based on wireless motility capsule data. Thus, the accuracy of the gastric emptying time may be lower than that of scintigraphic measures, as the solid capsule can be expelled from the stomach in the fasting state after emptying the solid meal [50]. However, gastric emptying times obtained using wireless motility capsules have been shown to correlate with 4 h scintigraphy measurements (sensitivity 0.87 and specificity 0.92) [50, 51]. The rationale for choosing the wireless motility capsules in the present study was to obtain a comprehensive evaluation of pan-enteric transit times in these individuals with multiregional dysmotility [1]. Seventh, the incorporation of a 2-week wash-out period between the study periods aimed to assess the efficacy of each treatment regimen independently, preventing potential carry-over effects from study period 1 that may interfere with study period 2. However, introducing a wash-out period also increases the risk of relative unblinding, particularly if the tVNS treatment demonstrated clinical effectiveness. Eighth, the observed symptom scores were generally lower than in previous studies investigating individuals with gastroparesis. Nonetheless, when only analysing participants with more than ‘mild symptoms’, the symptom changes were still comparable between groups. Lastly, the sham device only produces a humming sound, and the active device often induces twitching in the superficial facial muscles unilaterally. Thus, unintentional unblinding of participants during the trial is a potential risk.

In conclusion, the present randomised, sham-controlled, double-blind study provided no evidence of gastrointestinal symptom relief when applying short-term high-intensity or long-term medium-intensity cervical tVNS in individuals with diabetic gastroenteropathy compared with sham stimulation. Hence, tVNS in this format is probably not a recommendable adjuvant treatment to ease the burden of gastrointestinal symptoms in these individuals.

## Data Availability

The dataset generated during and analysed in the study is available upon reasonable request from the corresponding author.

## Abbreviations

CAN: Cardiac autonomic neuropathy
COMPASS: Composite autonomic symptoms score
CVT: Cardiac vagal tone
GCSI: Gastroparesis cardinal symptom index
GSRS: Gastrointestinal symptom rating scale
tVNS: Transcutaneous vagal nerve stimulation

## References

[1] Kempler P, Amarenco G, Freeman R (2011). Management strategies for gastrointestinal, erectile, bladder, and sudomotor dysfunction in patients with diabetes. Diabetes Metab Res Rev.

[2] Bharucha AE, Batey-Schaefer B, Cleary PA (2015). Delayed gastric emptying is associated with early and long-term hyperglycemia in type 1 diabetes mellitus. Gastroenterology.

[3] Bytzer P, Talley NJ, Leemon M, Young LJ, Jones MP, Horowitz M (2001). Prevalence of gastrointestinal symptoms associated with diabetes mellitus: a population-based survey of 15,000 adults. Arch Intern Med.

[4] Gaede P, Lund-Andersen H, Parving HH, Pedersen O (2008). Effect of a multifactorial intervention on mortality in type 2 diabetes. N Engl J Med.

[5] Acosta A, Camilleri M (2015). Prokinetics in gastroparesis. Gastroenterol Clin North Am.

[6] Bharucha AE, Kudva YC, Prichard DO (2019). Diabetic gastroparesis. Endocr Rev.

[7] McCallum RW, Snape W, Brody F, Wo J, Parkman HP, Nowak T (2010). Gastric electrical stimulation with Enterra therapy improves symptoms from diabetic gastroparesis in a prospective study. Clin Gastroenterol Hepatol.

[8] McCallum RW, Dusing RW, Sarosiek I, Cocjin J, Forster J, Lin Z (2010). Mechanisms of symptomatic improvement after gastric electrical stimulation in gastroparetic patients. Neurogastroenterol Motil.

[9] Azpiroz F, Malagelada JR (1987). Importance of vagal input in maintaining gastric tone in the dog. J Physiol.

[10] Bonaz B, Sinniger V, Pellissier S (2016). Vagal tone: effects on sensitivity, motility, and inflammation. Neurogastroenterol Motil.

[11] Zhang Y, Lu T, Dong Y, Chen Y, Chen JDZ (2021). Auricular vagal nerve stimulation enhances gastrointestinal motility and improves interstitial cells of Cajal in rats treated with loperamide. Neurogastroenterol Motil.

[12] Frokjaer JB, Bergmann S, Brock C (2016). Modulation of vagal tone enhances gastroduodenal motility and reduces somatic pain sensitivity. Neurogastroenterol Motil.

[13] Brock C, Brock B, Aziz Q et al (2017) Transcutaneous cervical vagal nerve stimulation modulates cardiac vagal tone and tumor necrosis factor-alpha. Neurogastroenterol Motil 29(5). 10.1111/nmo.12999 10.1111/nmo.12999 27957782

[14] Frangos E, Komisaruk BR (2017). Access to vagal projections via cutaneous electrical stimulation of the neck: fMRI evidence in healthy humans. Brain Stimul.

[15] Yap JYY, Keatch C, Lambert E, Woods W, Stoddart PR, Kameneva T (2020). Critical review of transcutaneous vagus nerve stimulation: challenges for translation to clinical practice. Front Neurosci.

[16] electroCore (2018) Instructions for use for gammaCore sapphire. Available from https://www.gammacore.com/wp-content/themes/gammacore-p2/pdf/gammacore-IFU.pdf. Accessed 1 September 2023

[17] Paulon E, Nastou D, Jaboli F, Marin J, Liebler E, Epstein O (2017). Proof of concept: short-term non-invasive cervical vagus nerve stimulation in patients with drug-refractory gastroparesis. Frontline Gastroenterol.

[18] Gottfried-Blackmore A, Adler EP, Fernandez-Becker N, Clarke J, Habtezion A, Nguyen L (2020). Open-label pilot study: Non-invasive vagal nerve stimulation improves symptoms and gastric emptying in patients with idiopathic gastroparesis. Neurogastroenterol Motil.

[19] Arora Z, Parungao JM, Lopez R, Heinlein C, Santisi J, Birgisson S (2015). Clinical utility of wireless motility capsule in patients with suspected multiregional gastrointestinal dysmotility. Dig Dis Sci.

[20] Farmer AD, Pedersen AG, Brock B (2017). Type 1 diabetic patients with peripheral neuropathy have pan-enteric prolongation of gastrointestinal transit times and an altered caecal pH profile. Diabetologia.

[21] Lartigue S, Bizais Y, Des Varannes SB, Murat A, Pouliquen B, Galmiche JP (1994). Inter- and intrasubject variability of solid and liquid gastric emptying parameters. A scintigraphic study in healthy subjects and diabetic patients. Dig Dis Sci.

[22] Okdahl T, Bertoli D, Brock B (2021). Study protocol for a multicentre, randomised, parallel group, sham-controlled clinical trial investigating the effect of transcutaneous vagal nerve stimulation on gastrointestinal symptoms in people with diabetes complicated with diabetic autonomic neuropathy: the DAN-VNS Study. BMJ Open.

[23] Revicki DA, Rentz AM, Dubois D (2004). Gastroparesis Cardinal Symptom Index (GCSI): development and validation of a patient reported assessment of severity of gastroparesis symptoms. Qual Life Res.

[24] Kulich KR, Madisch A, Pacini F (2008). Reliability and validity of the Gastrointestinal Symptom Rating Scale (GSRS) and Quality of Life in Reflux and Dyspepsia (QOLRAD) questionnaire in dyspepsia: a six-country study. Health Qual Life Outcomes.

[25] Gulichsen E, Fleischer J, Ejskjaer N, Eldrup E, Tarnow L (2012). Screening for diabetic cardiac autonomic neuropathy using a new handheld device. J Diabetes Sci Technol.

[26] D'Amato C, Greco C, Lombardo G (2020). The diagnostic usefulness of the combined COMPASS 31 questionnaire and electrochemical skin conductance for diabetic cardiovascular autonomic neuropathy and diabetic polyneuropathy. J Peripher Nerv Syst.

[27] Casellini CM, Parson HK, Richardson MS, Nevoret ML, Vinik AI (2013). Sudoscan, a noninvasive tool for detecting diabetic small fiber neuropathy and autonomic dysfunction. Diabetes Technol Ther.

[28] Greco C, Di Gennaro F, D'Amato C (2017). Validation of the Composite Autonomic Symptom Score 31 (COMPASS 31) for the assessment of symptoms of autonomic neuropathy in people with diabetes. Diabet Med.

[29] Rentz AM, Kahrilas P, Stanghellini V (2004). Development and psychometric evaluation of the patient assessment of upper gastrointestinal symptom severity index (PAGI-SYM) in patients with upper gastrointestinal disorders. Qual Life Res.

[30] Sarosiek I, Selover KH, Katz LA (2010). The assessment of regional gut transit times in healthy controls and patients with gastroparesis using wireless motility technology. Aliment Pharmacol Ther.

[31] Wang YT, Mohammed SD, Farmer AD (2015). Regional gastrointestinal transit and pH studied in 215 healthy volunteers using the wireless motility capsule: influence of age, gender, study country and testing protocol. Aliment Pharmacol Ther.

[32] Boulton AJ, Vinik AI, Arezzo JC (2005). Diabetic neuropathies: a statement by the American Diabetes Association. Diabetes Care.

[33] Spallone V (2019). Update on the impact, diagnosis and management of cardiovascular autonomic neuropathy in diabetes: what is defined, what is new, and what is unmet. Diabetes Metab J.

[34] Wegeberg AM, Lunde ED, Riahi S (2020). Cardiac vagal tone as a novel screening tool to recognize asymptomatic cardiovascular autonomic neuropathy: aspects of utility in type 1 diabetes. Diabetes Res Clin Pract.

[35] Farmer AD, Coen SJ, Kano M (2014). Normal values and reproducibility of the real-time index of vagal tone in healthy humans: a multi-center study. Ann Gastroenterol.

[36] Nonis R, D'Ostilio K, Schoenen J, Magis D (2017). Evidence of activation of vagal afferents by non-invasive vagus nerve stimulation: an electrophysiological study in healthy volunteers. Cephalalgia.

[37] Muthulingam JA, Hansen TM, Olesen SS, Drewes AM, Frøkjær JB (2022). Two-week cervical vagus nerve stimulation in chronic pancreatitis patients induces functional connectivity changes of limbic structures. Neuromodulation.

[38] Steidel K, Krause K, Menzler K (2021). Transcutaneous auricular vagus nerve stimulation influences gastric motility: a randomized, double-blind trial in healthy individuals. Brain Stimul.

[39] Teckentrup V, Neubert S, Santiago JCP, Hallschmid M, Walter M, Kroemer NB (2020). Non-invasive stimulation of vagal afferents reduces gastric frequency. Brain Stimul.

[40] Müller SJ, Teckentrup V, Rebollo I, Hallschmid M, Kroemer NB (2022). Vagus nerve stimulation increases stomach-brain coupling via a vagal afferent pathway. Brain Stimul.

[41] Farmer AD, Strzelczyk A, Finisguerra A (2020). International consensus based review and recommendations for minimum reporting standards in research on transcutaneous vagus nerve stimulation (version 2020). Front Hum Neurosci.

[42] Kaut O, Janocha L, Weismüller TJ, Wüllner U (2019). Transcutaneous vagal nerve stimulation improves gastroenteric complaints in Parkinson's disease patients. NeuroRehabilitation.

[43] Desai A, O'Connor M, Neja B (2018). Reproducibility of gastric emptying assessed with scintigraphy in patients with upper GI symptoms. Neurogastroenterol Motil.

[44] Muthulingam JA, Olesen SS, Hansen TM, Brock C, Drewes AM, Frøkjær JB (2021). Cervical transcutaneous vagal neuromodulation in chronic pancreatitis patients with chronic pain: a randomised sham controlled clinical trial. PLoS One.

[45] Juel J, Brock C, Olesen SS (2017). Acute physiological and electrical accentuation of vagal tone has no effect on pain or gastrointestinal motility in chronic pancreatitis. J Pain Res.

[46] Travagli RA, Anselmi L (2016). Vagal neurocircuitry and its influence on gastric motility. Nat Rev Gastroenterol Hepatol.

[47] Silberstein SD, Mechtler LL, Kudrow DB (2016). Non-invasive vagus nerve stimulation for the acute treatment of cluster headache: findings from the randomized, double-blind, sham-controlled ACT1 study. Headache.

[48] Illigens BM, Gibbons CH (2009). Sweat testing to evaluate autonomic function. Clin Auton Res.

[49] Novak P (2019). Electrochemical skin conductance: a systematic review. Clin Auton Res.

[50] Cassilly D, Kantor S, Knight LC (2008). Gastric emptying of a non-digestible solid: assessment with simultaneous SmartPill pH and pressure capsule, antroduodenal manometry, gastric emptying scintigraphy. Neurogastroenterol Motil.

[51] Rao SS, Camilleri M, Hasler WL (2011). Evaluation of gastrointestinal transit in clinical practice: position paper of the American and European Neurogastroenterology and Motility Societies. Neurogastroenterol Motil.

